# Early Mini-Invasive Treatment of Persistent Cervical Dysplasia: Clinical Outcome and Psycho-Relational Impact

**DOI:** 10.3389/fsurg.2022.888457

**Published:** 2022-05-19

**Authors:** Francesco Plotti, Gianmarco Rossini, Fernando Ficarola, Carlo De Cicco Nardone, Roberto Montera, Federica Guzzo, Daniela Luvero, Silvia Fabris, Roberto Angioli, Corrado Terranova

**Affiliations:** ^1^Department of Gynecology, Campus Bio-Medico University Hospital Foundation, Rome, Italy; ^2^ASST of the Olona Valley, Department of Obstetrics and Gynecology, Varese, Italy; ^3^National Center for Control and Emergency Against Animal Diseases and Central Crisis Unit–Office III, Directorate-General for Animal Health and Veterinary Drugs, Italian Ministry of Health, Rome, Italy; ^4^Medical Statistics and Epidemiology Unit, Campus Bio-Medico University, Rome, Italy

**Keywords:** t-LEEP, HR-HPV clearance, L-SIL, mini-invasive treatment, anxiety and sexual function

## Abstract

**Introduction:**

After the diagnosis of L-SIL, 77. 3% had a persistent infection and anomalous Pap Test results. Many of these patients had highlighted psychological consequences such as anxiety, hypochondria, fear of cancer, and sexual problems. Several studies suggested that the clearance of HR-HPV infection could be accelerated by cervical excisional procedures, especially in L-SIL. In consideration of the psychological implications for HPV infection and related dysplasia in patients with CIN1 at PAP-smear and HR-HPV positivity at least for 6 months, we decided to plan a prospective study where we tried to anticipate excisional cervical using a minimally invasive treatment: thin loop electrosurgical excision procedure (t-LEEP). This study aims to analyze the clearance of HR-HPV after 6 and 12 months, clinical outcomes related to t-LEEP, and the psycho-relational impact at 12 months after t-LEEP.

**Materials and Methods:**

We enrolled patients with the diagnosis of L-SIL at PAP-smear and HR-HPV positivity with a persistent CIN 1 (at least for 6 months), confirmed by cervical biopsy. All enrolled patients underwent t-LEEP. We followed prospectively and performed for all patients the HPV DNA test at 6 (T1) and 12 months (T2) and STAI-Y and FSFI scores at T0 and T2.

**Results:**

We prospectively enrolled 158 patients, 22 are excluded for the established criteria. Patients with HR-HPV and CIN 1 lesions treated with t-LEEP had an overall clearance of 83.8% at T2. In subgroups analysis at T2, we had a regression: in smoker 71.8%, in contraceptive users 69.5%, in patients aged <25 years 100%, aged 25–30 years 85%, aged 30–35 years 94.4%, aged 35–40 years 92%, and aged ≥40 years 89.1%, in HPV-16 96.4%, in HPV-53 89.5%, in HPV-18 87.5%, in HPV-31 86.6%, and in coinfected 3.5%. STAI-Y and FSFI after t-LEEP (T2) were statistically significant, reducing anxiety status and improving sexual function.

**Conclusion:**

Based on these results, the use of t-LEEP in patients with persistent CIN 1 and HPV-HR at least for 6 months could be useful for accelerating HPV-HR clearance, in particular, for a subpopulation patient with an increased risk of progression and/or patients with psychological and sexual consequences of carrying an HR-HPV infection.

## Introduction

High risk–human papillomavirus (HR-HPV) infection is associated with cervical intraepithelial neoplasia and its persistence is considered essential for the progression of cervical precancer to invasive cervical cancer (CC) (1, 2). The epidemiological research has confirmed that HR-HPV especially 16, 18, but also numerous genotypes are strongly associated with carcinogenesis and onset of CC (3, 4).

CIN grade 1 is a common cervical precancer, especially in young women who are sexually active. CIN 1 cervical precancer, often, has an overlap with low-risk HPV (LR-HPV) infection. In a recent study in the literature based on 2 years of follow-up data after diagnosis of low-grade cervical lesions with prior low-grade cytology, it was found that 22.7% of the patients experienced cytologic regression in the first year. Persistently altered cytology to PAP-smear was valued as 77.3% at 1st year of follow-up, in particular among women aged > 35 years, regression rates were lower than in younger people with an average persistence of ~80% after 28.8 months of follow-up (5, 6). The recent ASCCP guidelines in 2019 recommend a conservative follow-up of CIN 1 by colposcopy and cytology or HPV testing at 1 year. If CIN 1 is diagnosed consecutively for 2 years, immediate treatment by the diagnostic excisional procedure is an acceptable treatment based on the patient's preference and after thorough counseling on the risks and benefits of surgical procedure (7). The numerous studies had shown how presenting an alteration to the PAP test can have an impact on the psychophysical health of women. In the literature, different problems resulting from the diagnosis of HPV and dysplasia were cited such as anger, impotence, sexual problems and consequent reproductive difficulties, anxiety, hypochondria, and consequent fear for CC, but also an altered perception of body image up to fear of being labeled by your partner (8–11). The psychological aspect had not been much considered in ASCCP guidelines. It has been seen that the women who did not eliminate HPV spontaneously had greater recurrence rates and greater risk of developing high-grade cervical precancer lesions.

Several studies in the literature suggested that clearance of high-risk HPV infection could be accelerated by cervical excisional procedures especially in low-grade cervical lesions, thus quickly eliminating the main factor of development of cervical cancer (12).

Thin loop electrosurgical excision procedure (T-LEEP) is a modified surgical treatment for cervical lesions (thinner than a traditional LEEP), in which a minimal amount of cervical tissue is excised using electrosurgery with no impact on pregnancy outcomes and minimal adverse effects (13).

In consideration of the psychological implications of HPV-related dysplasia in these patients such as anxiety, avoidance of sexual intercourse with their partner, or delay in the pregnancy planning, we decided to plan a prospective study where we decided to anticipate excisional cervical treatment using t-LEEP at 6 months in all patients with persistent HPV HR-related dysplasia confirmed by cervical biopsy at 6 months. Our study aims to analyze the clearance of HR-HPV in different populations after 6 and 12 months, clinical outcomes related to t-LEEP, and the psycho-relational impact at 12 months after t-LEEP.

## Materials and Methods

We evaluated for the enrollment of all patients referred to the Departments of Obstetrics and Gynecology and Gynecologic Oncology, Campus Bio-Medico University Hospital of Rome with a cytological diagnosis of Low-Grade Squamous Intraepithelial Lesion (L-SIL) at cervical Papanicolaou (Pap) smear and HR-HPV positivity with a persistent CIN 1 (at least for 6 months), confirmed by cervical biopsy. Inclusion criteria for enrolment were as follows: (1) age between 16 and 70 years; (2) histological diagnosis of CIN 1 persistent for at least 6 months confirmed by colposcopically-directed cervical biopsy; (3) detection of HR-HPV preoperatively on a cervical or vaginal swab; (4) LSIL at Pap-smear; and (5) informed consent obtained from the patients. Exclusion criteria included the following: (1) HIV or immunosuppression; (2) history of chronic illness; (3) pregnant or minimum of 6 months postpartum; (4) treatment for cervical disease or abnormal cytology within 18 months. For each patient enrolled, we recorded the following: age, menopausal status, smoking status, the desire of pregnancy, parity, utilization of combined oral contraception (COC), and previous cervical surgical procedure.

All enrolled patients underwent different questionnaires to examine anxiety state and sexual function after enrollment (T0).

- State-Trait Anxiety Inventory for Adults, Form Y-1 (STAI-S): It measures state anxiety and consists of 20 items; STAI is a validated test for measuring preoperative anxiety (14, 15). We used form Y of STAI-20 translated into Italian.
- Female Sexual Function Index (FSFI): It measures the female sexual functions and consists of 19 items. It has been developed for the evaluation of female sexual arousal disorder (FSAD) with six domains of sexual function: desire, arousal, lubrication, orgasm, satisfaction, and pain (16).

All enrolled patients underwent t-LEEP under deep sedation. T-LEEP is a surgical treatment for cervical lesions, in which a coin-shaped (1–2 mm from the outer edge of the cervical transformation zone, with an excision depth of 3–5 mm) of cervical tissue is excised using electrosurgery (13). All tissue samples obtained by t-LEEP were pathologically examined. Therefore, we recorded the dimension of the cervical specimen (length, width, and thickness) and post-operative complications by the standardized system for the registration of surgical complications, the Clavien-Dindo Classification (17). We performed the HPV DNA test at 6 months (T1) and 12 months (T2) and STAI-Y and FSFI scores at T2 for all patients included in the study.

The HPV clearance rate was evaluated for all patients enrolled at T1 and T2: we performed a subgroup analysis based on age, HPV genotype, utilizers of combined oral contraceptives (COC), and smoking status. HPV reinfection rate and mean time clearance were also calculated; therefore, we compared the results of STAI-Y and FSFI preoperatively (T0) and post-operatively (T2) for all patients included in the study, and then, we performed a subgroup analysis in cleared and non-cleared patients.

This study was conducted following the regulatory standards of Good Clinical Practice and the Declaration of Helsinki (1996) and was approved by the institutional review board. An exploratory descriptive analysis of patient's characteristics and main outcomes was performed. The distributive normality of the data was evaluated through the Shapiro–Wilk test. We analyzed the data related to sexual function and psychological function using the robust two-way mixed ANOVA test, evaluating the relationship between the scores obtained in the various questionnaires, clearance, and clearance time. All tests were evaluated with a 95% significance level, and a *p* < 0.05 was considered statistically significant.

## Result

**Figure 1:**
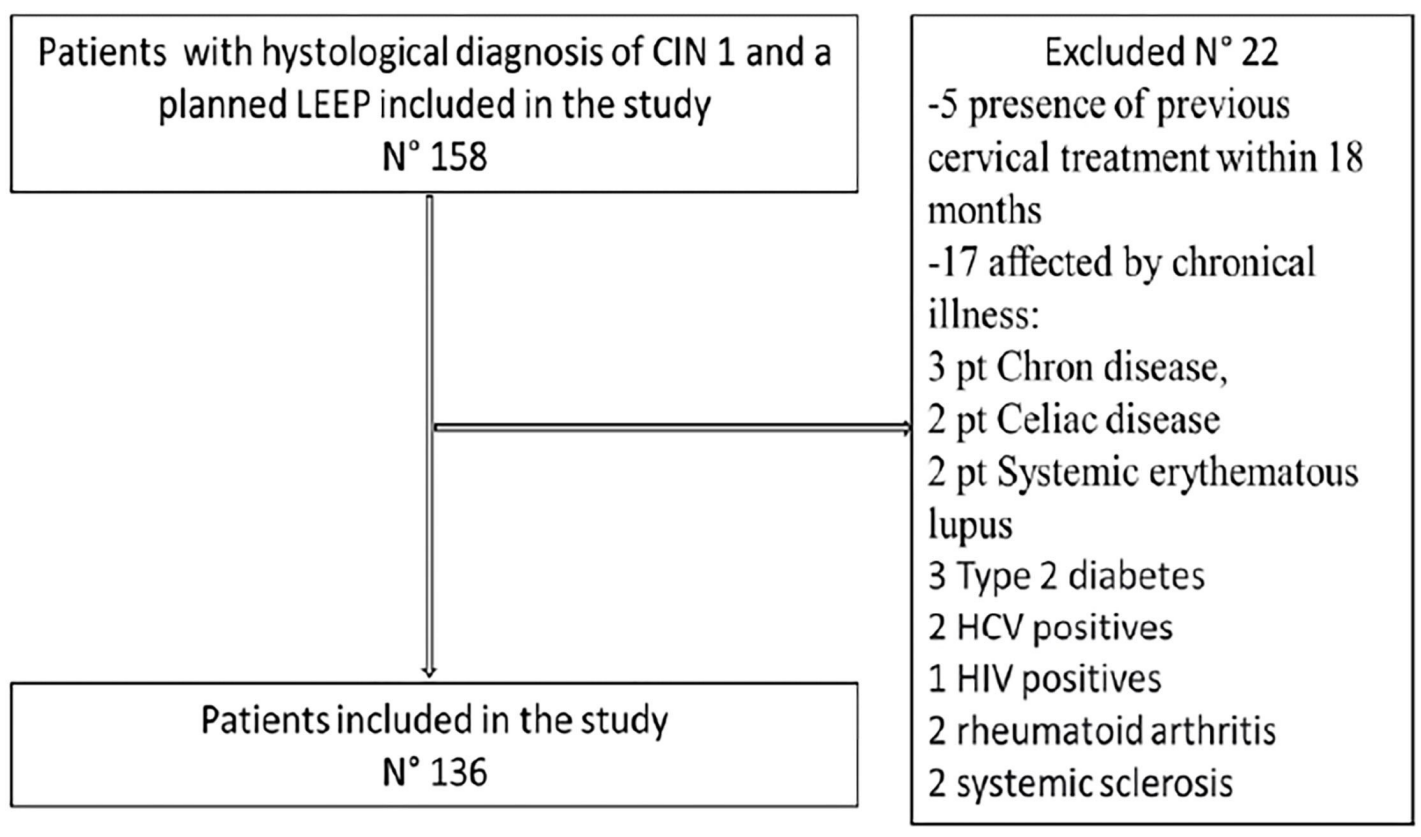
Flow diagram.

Between January 2015 and December 2019, we selected 158 patients referred to the Departments of Obstetrics and Gynecology and Gynecologic Oncology, Campus Bio-Medico University Hospital of Rome with a cytological diagnosis of Low-Grade Squamous Intraepithelial Lesion (L-SIL) at cervical Pap-smear and HR- HPV positivity with a persistent CIN 1 (at least for 6 months), confirmed by cervical biopsy. Of these, 5 were excluded for the presence of previous cervical treatment within 18 months and 17 were affected by chronic illness (3 patients Chron's disease, 2 Celiac disease, 2 systemic erythematous lupus, 3 type 2 diabetes, 2 HCV positives,1 HIV positives, 2 rheumatoid arthritis, and 2 systemic scleroses). At the end of enrollment, 136 patients were included in this study (Figure 1). The mean age of patients was 39.4 (range 20–72 years), patients in menopausal status were 22 (16.1%); smoker patients were 39 (28.6%); patients with maternal desire were 61 (44.8%); patients with parity > 1 before t-LEEP were 60 (44.1%), patients utilizers of COC were 46 (33.8%), and patients with previous cervical procedures were 20 (14.7%). A number of 9 patients were vaccinated for HPV on 136 patients 6.6% at enrollment. HPV DNA preoperative test showed an overall prevalence of (56/136) 41.1% for genotype 16, (19/136) 13.9% for genotype 53, (16/136) 11.7% for genotype 18, (15/136) 11.0% for genotype 31, (10/136) 7% for other genotypes (which includes HR-HPV 33, 35, 39, 45, 51, 52, 56, 58, 59, 66, 68, 70, 73, 82), and (20/136) 14.7% of coinfected patients with HR-HPV. Preoperative (T0) STAI-Y mean score was 58.4; FSFI means score t T0 was 23.3 (superior to the cutoff representative of sexual dysfunction) (Table 1).

**Table 1:** Patients characteristics, histological characteristics and post-operative complication.

		**T-Group (*N* = 136)**
Age (year mean)		39.4
Post-menopause (%)		22 (16.1%)
Smoking (%)		39 (28.6%)
Pregnancy desire (%)		61 (44.8%)
Parity >1		60 (44.1%)
Combined oral contraception		46 (33.8%)
Patients with previous cervical surgery		20 (14.7%)
**HPV-DNA (T0)**		
	16	56 (41.1%)
	53	19 (13.9%)
	18	16 (11.7%)
	31	15 (11.0%)
	Others	10 (7.0%)
	Coinfected with HR-HPV	20 (14.7%)
**STAI-Y (T0)**	Mean	58.4
**FSFI (T0)**	Mean	23.3
**Histological**		
CIN 1		132 (97%)
CIN 2/3		4 (3%)
**Mean dimension of cervical specimen**		
Width		9.5 mm
Length		14.8 mm
Thickness		3.6 mm
**Clavien–dindo classification of post-operative complications**		
Grade I		11 (8%)
Grade II		0 (0%)
Grade III		0 (0%)
Grade IV		0 (0%)
Grade V		0 (0%)

All patients consequently underwent t-LEEP under deep sedation. The mean thickness of the specimen of t-LEEP was 3.6 mm (range 1–10 mm), with a medium length of 14.8 mm (range 3–20 mm) and a medium width of 9.5 mm (range 2–18 mm). Histological examination of the t-LEEP specimen showed 132 patients with a diagnosis of CIN 1 (97%) and 4 patients with a diagnosis of CIN 2 (3%), and margins of the histological specimen were free from cervical dysplasia (Table 1). These patients underwent follow-up with colposcopy according to the ASCCP guidelines. If the patients had positive margins, they would have undergone a new conization surgery outside the study.

Post-operative complications were classified by Cleaven-Dindo classification. Only 11 patients (8%) reported Grade-1 complications (9 patients required post-operative analgesics and 2 patients required antiemetics) and no patients reported worse complications (Table 1).

The cumulative overall clearance rate of HPV-HR infection at T1 and T2 was 96 (70.5%) and 114 (83.8%), respectively. The total persistence at T1 and T2 was 29.5% and 16.2%, respectively (Table 2).

**Table 2:** Clearance subgroups.

**Clearance subgroups**	**HR-HPV clearance**	**HR-HPV clearance**
	**T1 (6 months)**	**T2 (12 months)**
**Overall clearance**		
**Cases**	96/136 (70.5%)	114/136 (83.8%)
**HPV clearance genotype**		
16 (56 pts)	51/56 (91.1%)	54/56 (96.4%)
53 (19 pts)	16/19 (84.2%)	17/19 (89.5%)
18 (16 pts)	11/16 (69%)	14/16 (87.5%)
31 (15 pts)	10/15 (66%)	13/15 (86.6%)
Others (10 pts)	6/10 (60%)	9/10 (90%)
Coinfected (20 pts)	2/20 (1%)	7/20 (3.5%)
Total (136 pts)	96/136 (70.5%)	114/136 (83.8%)
**Smoking patients**		
Smoking patients group (39 pts)	26/39 (66.7%)	28/39 (71.8%)
No-smoking patients group (97 pts)	70/97 (72.2%)	85/97 (87.6%)
**COC users**		
COC users group (46 pts)	28/46 (60.9%)	69.5% (32/46)
COC non-users group (91 pts)	64/91 (70.3%)	87.9% (80/91)
**Stratified by age**		
<25 (10 pts)	9/10 (90 %)	10/10 (100 %)
25–30 (19 pts)	14/19 (73.6%)	17/19 (89.4 %)
30–35 (18 pts)	14/18 (77.8 %)	17/18 (94.4%)
35–40 (25 pts)	15/25 (60 %)	23/25 (92%)
>40 (64 pts)	44/64 (73.3 %)	57/64 (89.1%)
Total	96/136	114/136

*Pts, patients; COC, Combined oral contraceptive*.

In subgroup analysis, we found that clearance rate is differently correlated with the genotype of HPV. In fact, the clearance at T1 was 91.1% for HPV16, 84.2% for HPV53, 69% for HPV 18, 66% for HPV31 60% for other types of HR HPV, and 1% for coinfected patients with different serotypes of HPV. This ratio increased at T2: 96.4% for HPV16, 89.5% for HPV53, 87.5% for HPV18, 86.6% for HPV31, 90% for other types of HR HPV, and 3.5% for coinfected patients with different genotype of HPV (Table 2).

In subgroup analysis for HPV clearance in smoking patients at T1, the clearance rate of the 39 smoking patients was 66.7% (26/39) and of the 97 non-smoking patients was 72.2% (*n* = 70/97). At T2, the clearance rate of smokers was 71.8% (28/39) compared to 87.6% (85/97) (Table 2).

In subgroup analysis for HPV clearance in patient users of COC: at T1, the clearance rate of the 46 patients users of COC was 60.9% (28/46) and of the 90 patients users of non-COC was 71.1% (*n* = 64/90). At T2, the clearance rate of COC users was 69.5% (32/46) compared to 87.9% (80/91) in non-users patients (Table 2).

When we analyzed the HPV clearance in different ages, we had a regression rate at T1 of 90% (9/10) in patients aged <25, 70% (14/19) in patients aged 25–30, 77.8% (14/18) in patients aged 30–35, 60 % (15/25) in patients aged 35–40, and 73.3% (44/64) in patients aged ≥40 and a regression rate at T2 of 100% in patients aged <25 (10/10), 85% (17/19) in patients aged 25–30, 94.4% (17/18) in patients aged 30–35, 92% (23/25) in patients aged 35–40, and 89.1% (57/64) patients aged ≥40 (Table 2).

Following cervical dysplasia diagnosis, we submitted the STAI-Y questionnaire to the 136 patients enrolled at T0. The main STAI-Y score at T0 was 58.4; we submitted the same form of STAI Y-20 at T2: the mean score decreased to 43.0 significatively (*p* < 0.0001) with a global reduction of anxiety status.

We performed therefore a subgroup analysis, and we observed that in cleared patients, mean STAI-Y T0 score was 59.2 and the T2 score was 40.0. The mean overall difference between T2 and T1 was 19.1 with a significant improvement in anxiety status (*p* < 0.00001).

In non-cleared patients, mean STAI-Y T0 score was 54.2 and the T2 score was 57.3. The mean overall difference was non-statistically significant (*p* = 0.18) with a persistent anxiety status. There is a significant two-way interaction between clearance status and time (*p* < 0.001). Moreover, we found a significant main effect in time (*p* < 0.001) (Table 3).

**Table 3:** Psycho-sexual state.

	**STAY-20 score T0**	**STAY-20 score T2**	***p*-value**
**Anxiety status stay-20-y**			
Overall	58.4	43.0	<0.00001
Cleared patients	59.2	40.0	<0.00001
Non-cleared patients	54.2	57.3	0.18
	**FSFI score T0**	**FSFI score T2**	***p*** **-value**
**Sexual function FSFI**			
Overall	23.3	27.30	<0.00001
Cleared patients	22.7	27.7	<0.00001
Non-cleared patients	26.4	25.2	0.23

Following cervical dysplasia diagnosis, we submitted the FSFI questionnaire to the 136 patients enrolled at T0. The main FSFI score at T0 was 23.3; we submitted the same form of FSFI at T2: the mean score increased to 27.30 (*p* < 0.0001) with a significant improvement of sexual function. We performed therefore a subgroup analysis, and we observed that in cleared patients, mean FSFI T0 score was 22.7 and the T2 score was 27.7. The FSFI score significatively increased (*p* < 0.00001), and it was superior to the cutoff indicated to define “sexual dysfunction” (<26.55). In non-cleared patients, mean FSFI T0 score was 26.4 and the T2 score was 25.2. At T2 mean score decreased but the difference between scores at T0 and T2 was non-statistically significant (*p* = 0.23) with a slight reduction of sexual function. There was a significant two-way interaction between clearance status and time (*p* < 0.001). Moreover, we found a significant main effect in time (*p* < 0.001) (Table 3).

During the 12-month follow-up, we collected collateral information on obstetric outcomes of patients undergoing t-LEEP. The result was: 62 had a desire for offspring after surgery (44.5%), 10 pregnancies occurred (in the study period), in 8 (80%) of which no adverse reactions were reported. The mean gestational age at birth was 39 + 2 weeks. None of them were hospitalized with a risk of preterm birth or threat of miscarriage. Two abortions occurred, one in the sixth week and the other in the eighth week of gestation. No obstetric outcomes beyond 12 months were reported in the study.

## Discussion

Our study aims to analyze the clearance rate of HR- HPV and clinical outcomes in women who have been treated by t-LEEP with histology of persistent CIN 1 (at least 6 months) confirmed by biopsy, and psycho-relational impact related to HPV clearance. The transformation zone is the one most susceptible to HPV infection and therefore predisposed to precancerous transformation with different degrees of dysplasia. It is characterized by metaplastic tissue, where there is a transition between the exocervical squamous epithelium that continues in the vaginal lining and the glandular tissue of the endocervix (18). This is the region where surgery is often done. Currently, there is no standard of care for HPV infection. ASCCP guidelines 2019 recommend a conservative follow-up of CIN 1 by colposcopy and cytology or HPV testing at 1 year. If CIN 1 is diagnosed on consecutive visits for at least 2 years, immediate treatment by the diagnostic excisional procedure is an acceptable option based on the patient's preference, after shared decision-making (7). Furthermore, the studies on women's positives for HPV infection and cytological HPV-related dysplasia have highlighted psychological consequences such as anxiety, fear of cancer, sexual problems, changes in body image, and difficulty in reproductive functions (8, 9).

So far, there is no reliable method to distinguish progressing from non-progressing CIN, therefore, neither validated medical nor conservative treatments for HPV infection: thus, the only validated treatment at this time remains surgical treatment that appears to be unpredictable for many patients. In our study, we propose to anticipate the cervical excisional treatment to clear rapidly HPV infection, thus avoiding psychological consequences related to HPV infection itself.

The different studies in the literature analyzed spontaneous viral clearance in HR-HPV-affected patients. Age, parity, cytology, viral load, and the presence of CIN 1 lesions at diagnosis were significantly associated with lesion progression, while human leucocyte antigen (HLA) DRB1^*^1301 seems to have a protective role in the pathogenesis of cervical cancer (19, 20). HPV vaccine may have a therapeutic effect in women with residual/recurrent CIN 1 or high-grade CIN (CIN 2–3) although further studies are needed to demonstrate this effect (21).

A good spontaneous regression rate of L-SIL cervical dysplasia is reported in the study by Moscicki et al. ranging from 61% at 12 months to 91% at 36 months (22).

In a cohort study of 44,102 women, the overall type-specific HR-HPV clearance rates in patients with borderline/mild dyskaryosis at 6- and 18-month clearance rates were 29 and 41% (23). Holowaty et al. in their cohort study with more than 17,000 women with CIN found that spontaneous regression of CIN 1 in 44% of patients within 2 years (24). Another cohort study analyzed 86 consecutive patients with CIN 1 and no surgical treatment. The rate of spontaneous regression at 24 months was overall 51.6%, compared with 34.7% in HPV16/18-positive cases and 59.9% in HPV16/18-negative cases (25). Different subpopulations had less capacity to clear infections such as HIV-positives, smoking women, women over 30, higher HPV viral burden, and more lifetime sex partners (26–28). A cohort of 2,065 women, aged 18–29 years, showed a spontaneous HR-HPV clearance rate of 61.2% with a follow-up of 12 months (29); in particular, younger patients had higher rates of regression and lower rates of progression of CIN. Among women aged 35–40 years, and > 40 years, regression rates were 27.3 and 24.9%, respectively, with a mean follow-up time during observational management of 28.8 months (6). Other studies in the literature observed a lower probability of clearing HPV infection among ever smokers compared with women who have never smoked. In addition, they detected a significant dose–response relationship with the increasing duration of years of smoking (17).

A systematic review of the literature that analyzed 24 studies worldwide with comparative data for 16,573 patients with CC and 35,509 without CC showed that there is an increased relative risk of CC in patients who use COCs and a decrease in relative risk after the cessation of the latter. Then, 10-year use of COCs cumulative incidence of invasive CC is estimated to increase from 3.8 to 4.5% in more developed countries and 7.3 to 8.3% in developing countries (30).

Kreimer et al. performed a study on 481 women infected with HPV, and the study reports that after LEEP, the regression rate of HPV infection is 74% for patients with HPV16, 81% for patients with HPV18, and a higher regression rate for infection of other HPV genotypes at 6 months of follow-up (31). HR-HPV infections cleared gradually after conization with clear resection margins on conization specimens (31). Another observational study of 110 women undergoing LEEP showed a regression rate of HPV of 80 and 88.2% at 12 and 18 months, respectively (12). A sub-analysis by genotype showed that persistence was greater for HPV16, with a persistence rate after LEEP of 72.7, 87.3, and 90% at 6, 12, and 18 months, respectively (12). A further sub-analysis for the HPV16-infected group at 6 months after LEEP treatment showed higher persistence in patients over 36.5 years of age (12).

Our study proposes to anticipate cervical excisional treatment at 6 months to clear rapidly HPV cervical infection; our data are comparable to data of the literature: cervical excisional treatment could accelerate HPV clearance with an overall clearance rate of 70.1% at 6 months and 83.2% at 12 months compared to spontaneous clearance rate that is around 30–50%. In particular, the proportion of cleared patients increases in subpopulations in which spontaneous clearance is usually reduced such as age > 35 years, smoker patients, and users of oral contraceptives.

Moreover, t-LEEP could be a low harming treatment with a very low percentage of complications. In our study, we showed only 8% classified as grade 1 and no major complication by Cleaven-Dindo Classification in 137 patients enrolled in the study. For the treatment of precancerous cervical lesions, both ablative methods (cervical cryotherapy, laser ablation) and LEEP excisional methods, cold cone) can be used effectively. Common complications of these procedures reported in the literature, including minor vaginal bleeding in the first 24 h after surgery which was 5.7% for cold cone, 5.4% for LEEP, 0.4% for cryotherapy; minor vaginal bleeding 24 h after surgery which was 8.3, 8.8, and 23.7% for cold cone, LEEP, and cryotherapy, respectively; cervical stenosis (cold cone 7%, LEEP 6.6%, cryotherapy 0.6%); pain (<30% for cold cone, LEEP, or cryotherapy) (32).

Although several studies have concluded that cervical excision procedures were associated with an increased risk of preterm birth, especially as excision depth increases, with a relative risk of 2.15 for excisional treatment >1.0 cm (33–37), others have found no such association after adjustment for potential confounding factors (38–41). T- LEEP is a minimally invasive procedure that removes the over-extending squamous and columnar epithelium and the naked vessel of the cervix. In our study, we showed that the mean thickness of the specimen is 3.6 mm, and then, the cervical function is likely minimal impaired in t-LEEP than in LEEP for CIN; consequently, the risk of preterm delivery in subsequent pregnancy may also be lower. However, further studies are needed to analyze the pregnancy outcomes in women with pregnancy desire.

Due to the sexually transmitted nature of HPV, psychosocial effects of HPV-linked diseases and abnormal cytology results have been demonstrated in the literature. The previous studies showed that even positive screen results for HPV might have psychosocial consequences. These effects could include a reduction of sexual desire or sexual satisfaction, feeling sexually unattractive, sexually anxious, or depressed (42). McCaffery et al. reported in their study that among the reactions to the HPV screening test, anxiety was the most common. Certainly, the anxiety reported by patients during the HPV screening test was related to the personal factors ranging from a history of previous infections to the sentimental situation, but also to sexual habits and socio-cultural standards (42).

Clarke et al. performed a study of 489 HPV-positive men and women, reporting that at the time of diagnosis of infection, the most frequent feelings were depression, anger, isolation, fear of rejection, shame, and guilt (43).

The psychological distress experienced by the patient was not limited to the screening test and HPV diagnosis to the screening test but extends to the other aspects that were corollary to this infection, such as fear of recurrent gynecological visits, apprehension of passing on HPV to your sexual partner and thus be negatively judged by the latter (44). All of this had a negative impact on the sexual wellbeing of the couple, negatively affecting the quality and quantity of sexual contacts (44).

Maggino et al. performed a study where on a group of 72 patients and how the diagnosis of HPV affects their quality of life and immediately after the moment of diagnosis, reporting how patients who tested positive for HPV had a higher level of anxiety, obsessions, compulsions, hygiene concerns, and other possible unlikely infections (44). An impact on sexual health was confirmed by another British study, which reports an increase in sexual health concerns 6 months after HPV diagnosis in positive patients, compared to patients who did not screen or who were negative (45). Another descriptive study that always concerns the sexual health of patients positive for HPV reported that after diagnosis, it is recorded: dissatisfaction with sexual life in 22% of patients, problems in achieving orgasm in 22% of patients, and a reduction of the number of intercourses in 44% of patients (46).

The stigma of having an STI sometimes may negatively impact sexual self-image (feeling “dirty,” “contaminated”) and may affect sexual spontaneity or alter sexual activities (42, 47, 48).

Our study confirms that early clearance of HPV can improve the anxiety status of patients (shown by the significant improvement of the score of the questionnaire administered), thus improving the quality of life. Therefore, the awareness of an HPV-HR infection, with a greater risk of evolution toward a CC, would seem to have a negative role in reducing sexual desire. The different studies in the literature have shown a negative effect on sexual function after HPV infection. In a study of 72 patients tested for HPV, there were no significant differences in sexual satisfaction between women HPV positives and women HPV negatives after a follow-up of 6 to 12 months following HPV result (44). Concern about transmitting HPV to their partner was common, and some patients were also worried about the possibility of their partner re-infecting them limiting virus clearance and increasing the risk of cervical cancer (49, 50). Alay et al. performed a study on 80 women sexually active, who tested positive for the diagnosis of HPV-HR, reporting a statistically significant reduction in sexual desire. Furthermore, the same study reports a reduction in the FSFI score in patients positive for HPV16-18, compared to patients negative for these HPV genotypes. Another interesting result reported by this study is that in these women the changes in sexual function, there is no statistically significant difference between the patients with normal or altered cytology, whereas there is a significant difference between the patients with HPV16/18-positive and the HPV-negative for these genotypes (51). This study makes us understand how women aware of being positive for HR-HPV have a negative psychological impact, which affects sexual function, as compared to the patients with abnormal cytology results (51).

In our study, we found a better overall anxiety status and a better sexual function after cervical treatment with a statistically significant result. In subgroup analysis, it also analyzed anxiety status and sexual function in HPV-cleared patients and HPV-non-cleared patients. In our study, we found a significant decrease of anxiety status in HPV-cleared patients shown by the reduction of STAI-Y score after cervical treatment. Similarly, we found a better sexual function in cleared patients: FSFI score increased in cleared patients after treatment with a mean score of 27.74 representatives of normal sexual function, superior to the optimal cutoff score for differentiating women with and without sexual dysfunction (mean score >26.55) (52). In non-cleared patients, our study showed a slight worsening of anxiety status and sexual function as shown by STAI-Y scores and FSFI scores before and after cervical treatment. These results are comparable to the results reported in the literature for which sexual dysfunction was seldom impacted by conization (53), but it was more related to the status of the cervical dysplasia and HPV infection rather than surgical treatment at the cervical level (54).

In our study, we found that anticipating surgical cervical treatment at 6 months of persistent CIN 1 related to HR HPV could be a valid alternative to conservative treatment. In our study, we demonstrated that t-LEEP is a low harming treatment with only a few post-operative complications; t-LEEP can also accelerate clearance of HR-HPV with a CIN 1 lesions at 6 and 12 months with a clearance rate superior to spontaneous clearance as reported in the literature: in patients at greater risk of progression and low possibility of spontaneous regression such as aged > 35 years, smokers, contraceptive users can benefit from early cervical treatment. Patients who benefited from early cervical treatment with consequent viral clearance showed an improvement in their state of anxiety and their sexual function and therefore a better quality of life.

## Conclusion

Based on these results, the use of t-LEEP in patients with persistent CIN 1 and HPV-HR at least for 6 months could be useful for accelerating HPV-HR clearance, in particular, for a subpopulation patient with an increased risk of progression and/or patients with psychological and sexual consequences of carrying an HR-HPV infection.

We suppose that t-LEEP could not greatly impact pregnancy outcomes due to a limited cervical tissue excised. Although pregnancy outcomes need further investigations, we could propose t-LEEP to patients who have already satisfied pregnancy desires and mentioned risk factors for dysplasia progression.

## Data Availability Statement

The raw data supporting the conclusions of this article will be made available by the authors, without undue reservation.

## Ethics Statement

The studies involving human participants were reviewed and approved by Gynecology Campus Bio-Medico. The patients/participants provided their written informed consent to participate in this study.

## Author Contributions

FP, GR, FF, CD, RM, FG, DL, SF, RA, and CT: substantial contributions to conception and design. FP, GR, FF, SF, and CT: drafting and revising the article critically for important intellectual content. FP and FF: final approval of the version to be published. All authors read and approved the final version of the manuscript and have contributed to the protocol/project development, data collection, management, data analysis, and manuscript writing and editing.

## Conflict of Interest

The authors declare that the research was conducted in the absence of any commercial or financial relationships that could be construed as a potential conflict of interest. The reviewer SL declared a past co-authorship with the authors to the handling editor.

## Publisher's Note

All claims expressed in this article are solely those of the authors and do not necessarily represent those of their affiliated organizations, or those of the publisher, the editors and the reviewers. Any product that may be evaluated in this article, or claim that may be made by its manufacturer, is not guaranteed or endorsed by the publisher.
